# Supplementary material for: The impact of sociocultural contexts on the knowledge, attitudes, and practices of adults living with HIV/AIDS in Ethiopia towards metabolic syndrome risks: A descriptive phenomenology study using the PEN-3 model
Source: PLoS One. 2024 Aug 22;19(8):e0308891. doi: 10.1371/journal.pone.0308891 (PMC11340946; doi:10.1371/journal.pone.0308891)
Supplement: S1 Fig — (DOCX) [file pone.0308891.s001.docx]

S1. Fig. Adapted studys conceptual framework,, demonstrates the emergence of KAP through PEN-3 domains, PLWHs, and Gedeo-Zone, in Sothern-Ethiopia,.([23](#_ENREF_23))([24](#_ENREF_24))
