# Supplementary material for: The impact of sociocultural contexts on the knowledge, attitudes, and practices of adults living with HIV/AIDS in Ethiopia towards metabolic syndrome risks: A descriptive phenomenology study using the PEN-3 model
Source: PLoS One. 2024 Aug 22;19(8):e0308891. doi: 10.1371/journal.pone.0308891 (PMC11340946; doi:10.1371/journal.pone.0308891)
Supplement: S3 Table — (DOCX) [file pone.0308891.s005.docx]

**S3.Table.WDC.Connection of the research question, participant responses, Conceptual Framework, PLWHs, Gedeo-Zone Southern Ethiopia, 2018**

| Question | Conceptual Framework | | Adequate | Inadequate |
| --- | --- | --- | --- | --- |
| How does personal knowledge form the basis of personal attitudes toward risk factors and methods for preventing MS and its risks? | Knowledge | | "Individuals, among people who live with HIV and who are often enraged and ill-tempered, are likely to be exposed to metabolic problems; such as blood pressure." (M1_FGD4). | "I have never heard of the cause and general indication of such health problem." (W2_FGD3). |
| Question | Conceptual Framework | | Favorable | Unfavorable |
| How does personal attitude form the basis of personal practices toward risk factors and methods for preventing MS risks? | Attitude | | "I believe that it is my responsibility to have knowledge about my health and to take care for myself because no health expert can control us to do or not to do so this is the individual's responsibility"(FP_Ind1) | Because of the attention given, I believe it should be for HIV." It is because... This sickness has just lately been identified in us... It's dreadful...These illnesses, on the other hand, have existed for a long time and continue to persist...They are not frightening (M3_FGD4). |
| Question | Conceptual Framework | | Adequate | Inadequate |
| How knowledge and attitude does gained about MS RISKS and its risk factors influence eating habits, physical exercise, smoking, and alcohol intake practices? | | Practice | "I say: eating vegetables, fruits, and light food items risks which do not discomfort us; as well as improving our living style and feeding system...help us to keep ourselves from such related health problems" (M8_FGD2). | “ I had previously heard that participating in sports can help us avoid many health hazards... However, I did not undertake it since I lacked the necessary strength" (M4_FGD2). |
