# Supplementary material for: The impact of sociocultural contexts on the knowledge, attitudes, and practices of adults living with HIV/AIDS in Ethiopia towards metabolic syndrome risks: A descriptive phenomenology study using the PEN-3 model
Source: PLoS One. 2024 Aug 22;19(8):e0308891. doi: 10.1371/journal.pone.0308891 (PMC11340946; doi:10.1371/journal.pone.0308891)
Supplement: Attachment — Submitted filename: Point by point respons letter to Ploseone.docx [file pone.0308891.s006.docx]

**Point by point letter**

**Manuscript Submission ID** : PONE-D-24-06838

**Title of the manuscript:** The Impact of Sociocultural Contexts on the Knowledge, Attitudes, and Practices of Adults Living with HIV/AIDS in Ethiopia towards Metabolic Syndrome Risks:A Descriptive Phenomenology Study Using the PEN-3 Model. We are willing to provide any additional information or documentation required by PLOS ONE to address their concerns regarding the consent process in our study.

Dear Editor, Here I presented the point by point responses in to three sections as follows:

I. **Section 1: Concerns raised by the editors and its team members about Journal Requirements:**

When submitting your revision, we need you to address these additional requirements.

1. Please ensure that your manuscript meets PLOS ONE's style requirements, including those for file naming. The PLOS ONE style templates can be found at https://journals.plos.org/plosone/s/file?id=wjVg/PLOSOne_formatting_sample_main_body.pdf and <https://journals.plos.org/plosone/s/file?id=ba62/PLOSOne_formatting_sample_title_authors_affiliations.pdf>
2. In the ethics statement in the Methods, you have specified that verbal consent was obtained. Please provide additional details regarding how this consent was documented and witnessed, and state whether this was approved by the IRB.
3. We note that your Data Availability Statement is currently as follows: "All relevant data is within the manuscript and its supporting information files."Please confirm at this time whether or not your submission contains all raw data required to replicate the results of your study. Authors must share the “minimal data set” for their submission. PLOS defines the minimal data set to consist of the data required to replicate all study findings reported in the article, as well as related metadata and methods (https://journals.plos.org/plosone/s/data-availability#loc-minimal-data-set-definition).For example, authors should submit the following data: - The values behind the means, standard deviations and other measures reported;- The values used to build graphs; - The points extracted from images for analysis.

Authors do not need to submit their entire data set if only a portion of the data was used in the reported study.If your submission does not contain these data, please either upload them as Supporting Information files or deposit them to a stable, public repository and provide us with the relevant URLs, DOIs, or accession numbers. For a list of recommended repositories, please see https://journals.plos.org/plosone/s/recommended-repositories. If there are ethical or legal restrictions on sharing a de-identified data set, please explain them in detail (e.g., data contain potentially sensitive information, data are owned by a third-party organization, etc.) and who has imposed them (e.g., an ethics committee). Please also provide contact information for a data access committee, ethics committee, or other institutional body to which data requests may be sent. If data are owned by a third party, please indicate how others may request data access.

**Responses given to the editors concerns by the author**

1. Dear Editors, Thank you for your feedback and additional requirements for the revision of our manuscript submitted to PLOS ONE. We have taken note of the style requirements for file naming and now we can ensure you that our revised manuscript adheres to these guidelines.
2. Regarding the participant consent, we would like to provide further details on this matter. In our study, we obtained informed consent from all participants prior to their involvement in the research. The consent process included a detailed explanation of the study objectives, procedures, and potential risks and benefits. Participants were given the opportunity to ask questions and clarify any doubts before providing their consent. Consent was obtained in a written form, with participants signing a consent form to indicate their voluntary participation in the study and this was approved by the IRB of AAU. All consent forms were securely stored and can be made available for verification if required. We hope that this additional information addresses the requirements regarding participant consent for the revision of our manuscript. Please let us know if any further clarification is needed. Thank you for your attention to these details.
3. We are ok with journal operating systems like a transparent peer review process, where we publish reviewers' reports with the article.

**Additional Editor Comments:**

- The manuscript is interested meanwhile, the reviewers have raised a number of points which we believe would improve the manuscript and may allow a revised version to be published in PLOS one.

**Responses given to the editors by the author**

- Dear Editors, Thank you for your feedback on our manuscript submitted to PLOS ONE.

We appreciate the interest shown in our study and would like to address the points raised by the reviewers to improve the manuscript for potential publication. Regarding the reviewers' comments, we are committed to making the necessary revisions to enhance the quality and relevance of our study. We carefully consider each point raised and incorporated changes that will strengthen the manuscript and align it with the standards of PLOS ONE. We acknowledge the importance of addressing the reviewers' feedback to ensure the completeness and accuracy of our research findings. We are dedicated to providing a revised version of the manuscript that reflects the valuable insights gained from investigating the impact of sociocultural contexts on the behavior of PLWHs towards metabolic syndrome and its associated risks in Southern Ethiopia., **Sincerely, Dr Girma Tenkolu Bune , Corresponding and main author**

II. **Section 2: Reviewers' comments and Reviewer's Responses to Questions**

|  | **Reviewers' comments:** | **Reviewer's Responses to Questions** |
| --- | --- | --- |
|  | 1. Is the manuscript technically sound, and do the data support the conclusions? The manuscript must describe a technically sound piece of scientific research with data that supports the conclusions. Experiments must have been conducted rigorously, with appropriate controls, replication, and sample sizes. The conclusions must be drawn appropriately based on the data presented. | Reviewer #1: Partly  Reviewer #2: Yes |
|  | 2. Has the statistical analysis been performed appropriately and rigorously? | Reviewer #1: N/A  Reviewer #2: N/A |
|  | 1. Have the authors made all data underlying the findings in their manuscript fully available?   The [PLOS Data policy](http://www.plosone.org/static/policies.action#sharing) requires authors to make all data underlying the findings described in their manuscript fully available without restriction, with rare exception (please refer to the Data Availability Statement in the manuscript PDF file). The data should be provided as part of the manuscript or its supporting information, or deposited to a public repository. For example, in addition to summary statistics, the data points behind means, medians and variance measures should be available. If there are restrictions on publicly sharing data—e.g. participant privacy or use of data from a third party—those must be specified. | Reviewer #1: Yes  Reviewer #2: No |
|  | 4. Is the manuscript presented in an intelligible fashion and written in standard English? PLOS ONE does not copyedit accepted manuscripts, so the language in submitted articles must be clear, correct, and unambiguous. Any typographical or grammatical errors should be corrected at revision, so please note any specific errors here. | Reviewer #1: Yes  Reviewer #2: Yes |

III. **Section 3: Reviewer#1: Comment to the authors and Responses given by the authors**

Please use the space provided to explain your answers to the questions above. You may also include additional comments for the author, including concerns about dual publication, research ethics, or publication ethics. (Please upload your review as an attachment if it exceeds 20,000 characters).

1. **Introduction Section:**
a) **Reviewer#1: Comment to the authors:**

The manuscript focuses on a pertinent issue in HIV care: PLWH knowledge, attitudes and practices (KAP) about Metabolic Syndrome.

A. .Introduction section lacked in-depth literature review on previous studies that examined the same topic and studies that used the PEN-3 model, and why the study was conducted. For example, are there other studies that have examined same topic among PLWH in other countries, or even in Ethiopia? What were the findings? What gaps and in what way does this study address them?
B. Why was the PEN-3 model selected as the analytical tool? Specifically, besides the PEN-3 model being developed for African contexts, in what ways (as shown in other studies) is it the appropriate model?
C. Also, there is the inconsistent use of the acronym: PLWH or PLWHA.

**Responses to Reviewer #1**

**Concerns Regarding the Introduction Section by the Authors**

**Dear Reviewer #1,**

We appreciate your emphasis on the importance of conducting a thorough literature review in the Introduction section. Your suggestion to delve deeper into previous studies relevant to the study topic and utilize the PEN-3 model has been taken into consideration. Please find below the responses addressing the concerns raised in the Introduction sections listed as A, B, and C.

A. In response to the feedback provided regarding the “A” section of Introduction section of our manuscript, we have taken into consideration the suggestions and made revisions to address the perceived shortcomings. The study presented in our paper utilizes the PEN-3 model as a theoretical framework to explore the sociocultural factors associated with metabolic syndrome among people living with HIV (PLWH).

To provide a more comprehensive overview of previous studies that have utilized the PEN-3 model, we have examined a selection of four research articles that have employed this theoretical foundation. These articles were chosen after a thorough review of the literature, which included the screening of a total of 13,705 global studies, with a focus on studies conducted in Africa, particularly in Sub-Saharan Africa and Ethiopia.

Our revised Introduction now includes a detailed discussion on how the PEN-3 model has been applied in various global studies focusing on communicable and non-communicable health issues. We have highlighted examples where the model has been used to understand sociocultural contexts related to obesity, diabetes, HIV/AIDS, cancer, smoking, and dietary habits among marginalized populations. Additionally, we have emphasized the importance of the model in developing context-specific interventions to address health challenges effectively.

Furthermore, we have provided insights into the methodological approaches used in studies that have employed the PEN-3 model, including qualitative, quantitative, and mixed methods. Specifically, we have discussed how the model has been utilized to explore the intersection of cultural contexts with health issues, with a particular focus on HIV/AIDS and non-communicable diseases.

We have also expanded on previous research that has applied the PEN-3 model in Africa and Sub-Saharan Africa, emphasizing the diverse range of health outcomes that have been explored using this framework. Examples include studies on cancer screening, hypertension, HIV/AIDS self-testing, and diabetes management, among others.

In the context of Ethiopian studies, we have incorporated information on the number of articles focusing on cultural perspectives related to health issues, the methodological approaches used, and the topics that have been examined. Additionally, we have highlighted the gap in the literature concerning the integration of the PEN-3 model in studies related to metabolic syndrome among PLWHs in Ethiopia. Thank you for the feedback, which has helped us enhance the clarity and depth of our Introduction section. For more clarity, please see the introduction part of the revised manuscript on page ____3____ to ____6_ Sincerely,

B. With respect to the issues of "why was the PEN-3 model selected as the analytical tool?" Once again, we value the feedback from the initial reviewer and aim to respond to the raised concerns regarding its use in our study in the following manner:

The decision to employ the PEN-3 model in this research, as opposed to other cultural frameworks, is grounded in four primary reasons:

**The PEN-3 model is specifically tailored for African contexts, offering a unique perspective to identify culturally significant factors.** Introduced in 1989, this model is instrumental in exploring individuals' attitudes, knowledge, and behaviors within their cultural milieu, particularly pertinent for examining public health issues from a sociocultural standpoint. Previous research has successfully utilized the PEN-3 model to analyze public health behaviors, providing a framework that integrates sociocultural contexts and pertinent factors into interventions. Originating from African contexts, notably East Africa and Ethiopia, it provides insight into public health challenges, aiding in recognizing influential factors in resource-limited settings. This model encourages the exploration of positive, negative, and distinct viewpoints, promoting dialogue and collaboration between researchers and communities to identify shared and differing perspectives. Comprising three key domains - cultural identity, relationships and expectations, and cultural empowerment - the PEN-3 model offers a comprehensive lens to understand how cultural dynamics interplay with public health issues. By delving into these domains, researchers can gain a holistic comprehension of the impact of cultural factors on health-related beliefs and behaviors, facilitating the development of targeted interventions aligned with the population's needs.

**The model accentuates the positive socio-cultural influences on health behaviors and outcomes.** It underscores the necessity to acknowledge and integrate these elements into culturally sensitive interventions, recognizing the significance of socio-cultural values in health-related initiatives. Understanding socio-cultural influences is pivotal in effectively addressing health issues. The PEN-3 model has been widely employed in global research across various health domains, including cancer, diabetes, HIV/AIDS, smoking, dietary choices, and obesity among marginalized communities referred to as "Others," contributing to over 100 studies globally. Its applications have paved the way for investigating how sociocultural contexts impact health behavior perceptions and for tailoring interventions to these contexts.

**The model serves as a useful tool for data collection, analysis, and interpretation.** Numerous scholarly works leverage the PEN-3 model to steer qualitative data collection and analysis techniques like categorization, cross-tabulation, and recontextualization, enhancing the understanding of health behaviors and the integration of culturally relevant elements into interventions.

**The model underscores the pivotal role of family and community in shaping behaviors.** Families and communities play a central role in influencing individuals' healthcare experiences and decisions, particularly in diverse healthcare settings. Family support is crucial in healthcare, emphasizing the necessity for tailored interventions to enhance care and reduce stigma for individuals, such as those affected by HIV/AIDS. Within the PEN-3 framework, importance is placed on community perspectives through qualitative inquiries, aiming to uncover perceptions of metabolic issues, knowledge of risk factors, and attitudes and behaviors concerning prevention and control. In addition to its African roots, these rationales provide further insights into the preference for this model. We have endeavored to address these critiques in line with the reviewers' feedback. Please refer to the updated introduction section of the manuscript for additional details. For more clarity, please see the introduction part of the revised manuscript on page ____4____ to ____6_ starting from the third paragraphes upto the end.

**C. Regarding the inconsistent use of the acronym,** we recognize the importance of clarity and consistency in terminology. Following a review of the literature, we ensured uniformity in referring to adults living with HIV (PLWHs) throughout the manuscript.

1. **Methods section:**
   A. Fragmented in its current state. It should be organized for clarity and flow. Since the focus of the study is to examine sociocultural context, more information is needed about the study setting – location, languages spoken, etc. For example, Why Gedeo zone of Ethiopia? What makes that zone the location for the study?
   B. It is unclear what language the FG discussions were conducted in: English or local language? While there is reference in the result section about the use of local words to describe some things, it’s unclear whether this was the language used in data collection. Was the discussion questions in English or translated into the language of the community? This is necessary if the goal is to explore the sociocultural context as language is the conduit through which thoughts, feelings and perspectives are expressed, and it’s a product of the sociocultural context of where it is used.
   C. Also, there is the use of acronym not explained: DURH, WHC? On page 7, line 8: what was pretested? It’s not clearly stated.
   D. Though the author(s) included a table showing how the PEN-3 model was used, there is lack of depth in how the model was actually used. For example, was the PEN-3 model used in the development of FGD questions? Was it used for analysis of the data after the study was conducted?
   E. It seems overly simplified to use the PEN-3 model to aggregates items in the domains without thorough engagement of the tool-kit. In its current state of the data presentation, it conveys the idea that the model was used after the study was conducted to guide data “arrangement” not necessarily data analysis. That is, the PEN-3 model was used in organizing the data, and not used in guiding the data analysis. If this is the case, then it should be clearly stated. Data Analysis: How was the codebook generated? (page 9, line 3-4). As stated above, in its current state, the PEN-3 model was used to arrange the data not data analysis.

**Responses given to the Reviewer #1, concerns rose in the method section by the authors**

Dear Reviewer #1,

We appreciate your emphasis on the importance of conducting a thorough Methods Section. In response to feedback, we enhanced clarity and organization of the Methods section in the manuscript, including details about the study setting in the Gedeo zone of Ethiopia. The revised section addresses questions raised by reviewers on why this location was chosen for the study. Please find below the responses addressing the concerns raised in the Introduction sections listed as A, B, C, D, and E.

A. **About Fragmented flow:** The Method section now follows the journal's recommended presentation outline. Refer to the revised manuscript for a clear flow of ideas. For more clarity please see the revised manuscript method section on page ____6____ to the end.
B. **Regarding the Study Setting:** The study took place in the Gedeo zone of Southern Ethiopia, chosen for its unique sociocultural characteristics relevant to researching the influence of sociocultural contexts on the behavior of people living with HIV/AIDS (PLWHs) towards metabolic syndrome (MS) and associated risks. The diverse population in the Gedeo zone, with various cultural practices and languages such as "Amaharic" and "Gedeoffa," may impact health behaviors among PLWHs. To address this, we specified in the Methods the language used during data collection, particularly in FG discussions conducted in the local working language of the Gedeo zone. Effective communication was ensured by including team members proficient in both "Amaharic and Gedeo language.” **For more information, please refer to the method section, Sampling methods and procedures part in the subsection of** Setting **part on page 9 to 11.**
C. **Concerning Unexplained Acronyms:** In academic writing, clarity is key. Therefore, we already explained acronyms like DURH and WHC used in the manuscript. After reviewing the paper, we consistently referred to Dilla University Referral Hospitals (DURH) and Wonago Health Centers (WHC) throughout, ensuring readers' understanding by initially spelling out the full names followed by the acronyms in parentheses for subsequent mentions. **For more information, please refer to the method section, Sampling methods and procedures part in the subsection of** Setting **of data collector part part on page 10, starting line No one to eight.**
E. **About the PEN-3 model, it encompasses the cultural identity, relationship and expectation, and cultural empowerment domains.**

**The model was utilized in both pre and post data collection phases.** Prior to data collection, it guided the creation of Focus Group Discussion (FGD) questions. By integrating the PEN-3 model during question formulation, our aim was to pose culturally sensitive, contextually relevant questions in line with the study objectives. Following the data collection, the model was utilized to guide our analysis in three phases. In the initial phase, data capturing and transcription were conducted step by step, incorporating Dr. GirmaTenkolu (DGT) alone, while Sadat Mohamed (SM) manually used a pre-established codebook derived from a theoretical model and the Knowledge, Attitude, and Practice (KAP) conceptual frameworks. As mentioned earlier, the model constructs were employed for coding, categorizing, formulating subthemes and themes in this phase. It also facilitated the interpretation of word data and other data to uncover cultural nuances influencing participants' perspectives on metabolic syndrome risks. The model allowed for the exploration of positive, existential, and negative cultural factors that shape knowledge, attitudes, and practices in this context. Ultimately, its integration in question development and data analysis aimed for a comprehensive and culturally sensitive examination of sociocultural impacts on Ethiopian adults with HIV/AIDS. Its usage enhanced our findings, providing insights into the intricate interplay of culture, beliefs, and behaviors concerning metabolic syndrome risks.

**Concerning the statement, "Using the PEN-3 model to aggregate items in the domains without thoroughly engaging the tool-kit seems overly simplified.", we wish to clarify its central role in our study.** Despite perceptions of prioritizing data organization over analysis, the model deeply influenced our research from formulating questions to interpreting results. Regarding data analysis, the model was integral, categorizing participant responses on metabolic syndrome risk perceptions into its positive, existential, and negative cultural factors. This categorization informed a comprehensive codebook, aiding in identifying data patterns and themes. Additionally, the model guided interpretation of focus group discussion findings, revealing cultural influences on participants' knowledge, attitudes, and practices related to metabolic syndrome risks. Our methodology section in the revised manuscript now explicitly outlines how the PEN-3 model guided both data organization and analysis processes, demonstrating its significant impact on shaping study outcomes. For a detailed insight, we encourage a review of the updated methodology section.

2. **Results section:**

This needs to be seriously re-organized to focus on presenting the results of the study.

A. There is a mix-up with comments from the author and participants’ quotes making for lack of clarity. Participants’ quotes to support the points or themes should be presented clearly.
B. Also, there is confusing focus on the purpose of the study based on the comments/quotes of participants. Is the focus on Metabolic Syndrome or Multiple Sclerosis? The author needs to define the study focus, and should indicate if participants introduced a topic and/or idea “outside” of the focus of the study, like Multiple Sclerosis. So, it is unclear from the study what the sociocultural contexts of metabolic syndrome and NCDs are in the current state of the manuscript.
C. The use of words like “individuals living with HIV lack sufficient contextual knowledge and value system” (pg. 22) belies the purpose of the study. People’s value system is product of their sociocultural context and serves as a lens in interpreting their experiences (phenomenon).

**Responses given to the Reviwer#1 concerns rose on the Results section by the authors**

Dear Reviewer#1,

Thank you for reviewing our manuscript and providing valuable feedback. We appreciate your insights on the Results section of our article. We understand the need to re-organize the Results section to focus clearly on presenting the study findings. To enhance readability and coherence, we have revised the section as follows:

A. **Re-organization of Results Section:** We have restructured the results to distinguish between author comments and participants' quotes clearly. Participants' quotes are now presented in an organized manner supporting identified themes and key points. Each participant quote is aligned with relevant points or themes. **Findings are rearranged according to distinct themes or key points for a structured and coherent narrative.**
B. Clarification of Study Focus: Our study focuses on sociocultural settings for adults with HIV/AIDS in Ethiopia concerning metabolic syndrome and non-communicable diseases related lifestyle risks. We emphasize understanding how sociocultural contexts affect knowledge, attitudes, and practices related to metabolic syndrome risks. We have removed references to **Multiple Sclerosis,** as it was not relevant to our research.
C. **Clarity in Terminology and Purpose:** We have provided clearer explanations in the revised manuscript, focusing on how sociocultural contexts influence individuals' value systems regarding metabolic syndrome risks. These revisions aim to enhance the presentation of study results. We appreciate your feedback and are dedicated to meeting journal standards. Please refer to the Results section on pages p. 19 onwards for further details.
3. **Discussion section:**
A. Like the introduction, this section also lacks depth though the author included many references as to the use of the PEN-3 model.
B. Also, the lack of clarity in the results section, and the use of phrases that belies the purpose of the study, makes the discussion and conclusion section weak in achieving the goal of the study: sociocultural factors on KAP of PLWH.
C. In its current state, it did not show that the PEN-3 model was the theoretical basis for the study, neither did the study show “that sociocultural contexts have a significant role in the lack of awareness regarding risk factors and prevention methods for MS among PLWHs.

**Responses given to the Reviewer#1 concerns rose in the Discussion section by the authors**

Dear Reviewer #1,

Thank you for your feedback on our article. We appreciate the thoughtful assessment and would like to address the concerns you raised regarding the Discussion section.

A. **Enhancing Depth in Discussion Section:** In response to the feedback about the lack of depth, we have further integrated the findings with the theoretical underpinning of the PEN-3 model in the revised manuscript. By exploring the implications of sociocultural contexts on the knowledge, attitudes, and practices of PLWH towards metabolic syndrome risks, we aim to provide a more comprehensive discussion. For instance, we elaborate on how components of the PEN-3 model—Person, Extended Family, Neighborhood—the contextual knowledge influence the attitudes and practices of PLWHs in Ethiopia related to MS risks. The discussion section on pages p. 76 onwards for further details.
B. **Improving Clarity in Results Section:** We recognize the need for greater clarity in the Results section. In the revised manuscript, we have presented the results in a more coherent and focused manner, aligning them closely with the study's objectives. By revising the language to accurately reflect the study's purpose, we strengthen the connection between results and the overarching goal of examining sociocultural factors influencing the KAP of PLWH towards metabolic syndrome risks. For example, we highlight how sociocultural factors affect the awareness of MS risk factors and prevention methods among PLWHs in Ethiopia. Please refer to the Results section on pages p. 19 onwards for further details.
C. **Clarifying the PEN-3 Model as Theoretical Basis:** We understand the concern about clarity on the PEN-3 model as the theoretical basis for the study. In the revised manuscript, we provide a detailed explanation of how the PEN-3 model guided the research design, data collection, and analysis. We explicitly demonstrate the role of sociocultural contexts in affecting the awareness levels of MS risk factors and prevention methods among PLWHs. By incorporating specific examples from the data analysis, we aim to illustrate the direct link between the PEN-3 model and the study outcomes. We believe these revisions will enhance the quality and impact of the Discussion section and address your concerns. Thank you for your feedback, and we look forward to revising and resubmitting the manuscript for your consideration.
b) **Reviewer#2: Review Comments to the Author**

This study provides significant results that can guide future interventions in Ethiopia and In Africa in general. Here are some minor revisions needed:

A. The abstract should introduce the PEN-3 model
B. line 10 'Ethiopian studies' could be changed to ' studies in Ethiopia
C. The discussion should include a paragraph on how cultural identity has been shown to influence health behaviors in the Ethiopian context in both positive and negative ways
D. It should also include how cultural empowerment plays in the African context in general and in Ethiopia in particular.

**Responses given to the Reviewer#2 concerns rose in the Discussion section by the authors**

**Dear Reviewer #2,**

Thank you for your thoughtful review of our manuscript. We appreciate your positive feedback and recommendations for minor revisions to enhance the clarity and relevance of the study. We have carefully considered your suggestions and will incorporate them into the revised version of the article.

A. **Introduction of the PEN-3 Model in the Abstract:** We acknowledge the importance of introducing the PEN-3 model in the abstract to provide readers with a clear understanding of the conceptual framework used in the study. In the revised abstract, we included a brief overview of the PEN-3 model and its relevance to the investigation of sociocultural influences on knowledge, attitudes, and practices related to metabolic syndrome risks among adults living with HIV/AIDS in Ethiopia. **Please see the last line of the background in the abstract section for more justification.**
B. **Revision of 'Ethiopian studies' to 'studies in Ethiopia':** We appreciate the suggestion to change 'Ethiopian studies' to 'studies in Ethiopia' for clarity and accuracy. The modification was made in the manuscript to ensure consistency and precision in describing the research conducted within the Ethiopian context. **Once again I will invite the reviewer to evaluate the manuscript.**
C. **Inclusion of Cultural Identity Influence on Health Behaviors in Ethiopia:** Thank you for highlighting the significance of discussing how cultural identity influences health behaviors in the Ethiopian context. In the revised discussion section, we did our level best to incorporate a paragraph that explores the impact of cultural identity on health behaviors, highlighting both positive and negative influences observed in studies conducted in Ethiopia. **Please refer to the discussion section in the following two to four paragraphs starting on page 76 -79 for more confirmation.**
D. **Integration of Cultural Empowerment in the African and Ethiopian Context:** We agree with the suggestion to discuss the role of cultural empowerment in the African context and specifically in Ethiopia within the manuscript. In the revised version, we already included a section that explores the concept of cultural empowerment and its implications for health promotion and meaningful engagement within communities, particularly in the context of addressing metabolic syndrome risks among adults living with HIV/AIDS. Thank you for your valuable feedback, and we are committed to implementing these revisions to enhance the quality and relevance of our study. We look forward to resubmitting the article for your consideration. **For more reference, please take a look at it the revised manuscripts specific area where concerns raised like the discussion part , starting on page 83-84, starting the line No 18 up to the end. Regards!**
